# Hydrothermal synthesis of LaFeO_3_ nanoparticles adsorbent: Characterization and application of error functions for adsorption of fluoride

**DOI:** 10.1016/j.mex.2020.100786

**Published:** 2020-01-14

**Authors:** Mohammad Mesbah, Soudabeh Hamedshahraki, Shahin Ahmadi, Mostafa Sharifi, Chinenye Adaobi Igwegbe

**Affiliations:** a Young Researchers and Elite Club, Science and Research Branch, Islamic Azad University, Tehran, Iran; b Department of Biostatistic, Zabol University of Medical Sciences, Zabol, Iran; c Department of Environmental Health, Zabol University of Medical Sciences, Zabol, Iran; d Department of Chemical Engineering, Nnamdi Azikiwe University, Awka, Nigeria

**Keywords:** Hydrothermal Synthesis of LaFeO_3_ Nanoparticles Adsorbent: Characterization and Application of Error Functions for Adsorption of Fluoride, Fluoride, Lanthanum ferrite nanoparticles, Langmuir, Adsorption, Isotherm

## Abstract

The adsorption of fluoride from aqueous solution by lanthanum ferrite nanoparticles (LaFeO_3_ NPs) synthesized by the hydrothermal method has been investigated. This experimental study was conducted on a laboratory scale. The effects of various operating parameters such as pH (3–11), LaFeO_3_ NPs dosage (0.1–1.0 g/L), contact time (15–120 min), temperature (303–318 K), and initial concentration of fluoride (15–40 mg/L) on fluoride adsorption were studied. The results showed that under optimal conditions of fluoride concentration of 20 mg/L, pH of 5, LaFeO_3_ NPs dosage of 0.9 g/L, temperature of 308 K, and contact time of 60 min, maximum percentage removal of 94.75 % was obtained. The process of fluoride adsorption on LaFeO_3_ NPs was found to depend on the Freundlich adsorption and Koble–Corrigan isotherm models. The monolayer adsorption capacity of LaFeO_3_ NPs was 2.575 mg/g. The kinetic data fitted best into the pseudo-second-order model considering the values of the regression coefficients (r^2^) and error functions used. The thermodynamics study indicated that the adsorption process was exothermic (Δ*H*°< 0) and spontaneous (ΔG°< 0) in nature. It could be concluded that the synthesized LaFeO_3_NPs can be used as an effective adsorbent for fluoride ions removal from aqueous solutions.

## Graphical abstract

**Figure:**
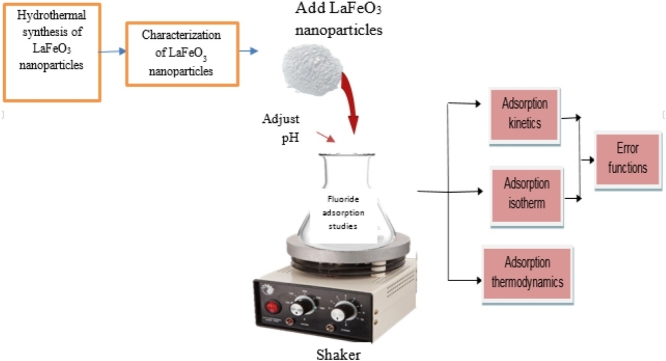

**Specification Table**

**Table:** 

Subject Area:	Environmental Science
More specific subject area:	Adsorption
Protocol name:	Hydrothermal Synthesis of LaFeO_3_ Nanoparticles Adsorbent: Characterization and Application of Error Functions for Adsorption of Fluoride
Reagents/tools:	Required reagents • Fluoride • Distilled water • Polyvinylpyrrolidone (PVP) coating agent • Iron salt [Fe(NO_3_).9H_2_O] • Lanthanum salt (La(NO_3_)_3_.6H_2_O) • sodium hydroxide (NaOH) • Ethanol (C_2_H_5_OH) • Hydrogen chloride (HCl) List of equipment • UV–visible spectrophotometer (Shimadzu Model: CE-1021-UK, Japan) • MIT65 pH meter • Philips diffract meter model PW1800 (The Netherlands) • Scanning electron microscope (Mira 3-XMU instrument capable of 700,000 x magnifications) • Fourier-transform infrared spectroscopy (JASCO 640 plus machine) • Spatula • Digital analytical balance • Mechanical shaker • Autoclave • 250 mL Erlenmeyer flasks • Digital thermometer • Oven • Centrifuge • Measuring cylinder
Experimental design:	The influence of pH, contact time, initial fluoride concentration, temperature and LaFeO_3_ NPs dose on fluoride adsorption process. Spectral properties of LaFeO_3_ NPs. Adsorption kinetic, isotherm and thermodynamic parameters were also presented.
Trial registration:	Not applicable
Ethics:	Not applicable

**Value of the Protocol**

**Table:** 

• The presented data established that LaFeO3 NPs can be applied for the removal of fluoride with great efficiency. • Data on the adsorption isotherm, kinetics, thermodynamics, and effect of process parameters were presented. • XRD, FE-SEM, and FTIR data for the LaFeO3 NPs were also provided. • The dataset will also serve as reference material to any researcher in this field.

## Description of protocol

Fluoride is one of the soluble ions in water resources that originate from natural and artificial sources including wastewater discharge of different industries and fluoride glass production industry [1]. It is also a natural element among minerals, geochemical sediments, and natural water systems, which enters the food chain through drinking water or feeding on plants. When its content in water is low, it should be added to water artificially. The presence of fluoride in water is essential to prevent dental decay [2]. On the other hand, if its content exceeds the desired level, it causes dental fluorosis and skeletal fluorosis [3]. This condition causes weakening of tooth and bone structure. It also declines growth and even in severe cases, it causes paralysis and death [4,5].

Today, various methods are used to remove organic and inorganic pollutants including absorption, biosorption, and adsorption by active alumina and manganese oxide coated with alumino along with various coagulators such as alum, ferric sulfate, ferro sulfate, ferric chloride, anionic, cationic, and non-ionic organic polymers [[6], [7], [8], [9]]. Physical adsorption is an efficient and economical method. Extensive research has been conducted on the adsorption of fluoride using different adsorbents including activated carbon [8]. It has also been proven that the adsorption process is a reliable treatment solution owing to minimum investment, convenient design and operation, and insensitivity to toxic compounds [9].

Recently various rare earth materials such as lanthanum, lanthanum modified activated alumina, lanthanum oxide, lanthanum impregnated green sand, cerium, and yttrium have been used as sorbents for the removal of fluoride from water [[10], [11], [12]]. Though lanthanum has got a good affinity for fluoride, there are some difficulties related to its use as an adsorbent. Recently, the magnetic properties of lanthanum ferrite nanoparticles (LaFeO_3_ NPs) have been extensively studied but the magnetic study of LaFeO_3_ NPs is rare [10]. Ant ferromagnetic nanoparticles always show unusual magnetic properties due to their finite-size effects, surface anisotropy effects, interface effects and shape anisotropy effect [[10], [11], [12]]. The nano-size of LaFeO_3_ NPs system has been majorly investigated as an alternative. Various types of LaFeO_3_ NPs can be synthesized by many methods such as sol-gel, co-precipitation, bull milling, sonochemical, and hydrothermal [10]. The hydrothermal method is one of the most powerful and widely used methods for the production of nanostructures; this has attracted a great deal of attention due to its simplicity and cost [13].

The main purpose of this study is to synthesize LaFeO_3_ NPs and investigate its effectiveness on the removal of fluoride from its aqueous solution. The impact of various operating factors such as contact time, LaFeO_3_ NPs dosage, pH, temperature and initial fluoride concentration on the fluoride adsorption process was studied to ascertain their optimum conditions. The adsorption kinetics, isotherm, and thermodynamics of the fluoride adsorption process on LaFeO_3_ NPs will also be studied. Error functions were also employed to compare the fit of adsorption isotherm and kinetic models in order to limit error between the predicted and experimental values.

## Materials and methods

### Preparation of LaFeO_3_ NPs

Lithium nanostructure was used with poly vinyl pyrrolidone (PVP) coating agent, and was then dissolved in distilled water using hydrothermal method with equal ratios of iron salt (Fe(NO_3_).9H_2_O) and lanthanum salt (La(NO_3_)_3_.6H_2_O) (that is, 0.2 g each was dissolved in 20 mL of distilled water and added to each other).

In another container, 0.5 g of PVP which had been dissolved in 40 mL of distilled water (at a temperature of 25 °C) was added to the reaction container. After vigorous stirring for 30 rpm, NaOH alkaline agent was added to the reaction container in order to raise the pH to 11. Next, the intended solution was transferred to an autoclave and placed inside an oven for 24 h at 200 ⁰C. Thereafter, the obtained solution was washed several times with distilled water and ethanol and then dried in an oven for 30 min at 343 K.

### Characterization of nanometer-sized LaFeO_3_ NPs

X-ray diffraction (XRD) patterns on the LaFeO_3_ NPs were taken by means of a Philips diffract meter model PW1800 (The Netherlands). The X-ray source was Cukα with 1.541 nm wavelength. Scanning electron microscopy (Mira 3-XMU instrument capable of 700,000 x magnifications) was used to study the morphology of the LaFeO_3_ NPs. Fourier-transform infrared spectroscopy (FT-IR) analysis of LaFeO_3_ NPs was done using a JASCO 640 plus machine (4000−400 cm^−1^) at room temperature to determine the functional groups presently involved in the fluoride adsorption process.

### Batch adsorption experiments

The effects of different parameters such as pH (3, 5, 7, 9 and 11), contact time (15, 30, 60, 90, and 120 min), temperature (303, 308, and 318 K), initial fluoride concentration (15, 20, 25, 30 and 40 mg/L) and LaFeO_3_ NPs dosage (0.1, 0.5, 0.7, 0.9 and 1 g/L) on the fluoride adsorption process were studied. A specified amount of adsorbent (LaFeO_3_ NPs) was added to Erlenmeyer flasks containing 100 mL of the solutions to be treated having different concentrations of fluoride. The pH of the solution was adjusted by adding 0.1 N HCl or 0.1 N NaOH. The flask with its contents was stirred for a specified time at 150 rpm. The resulting solution was centrifuged and the supernatant was analyzed for the residual fluoride concentration. The initial and final (or residual) fluoride concentrations in the solutions were determined using a UV–vis spectrophotometer (Shimadzu Model: CE-1021-UK, Japan) at a wavelength of maximum absorbance (*λ_max_*) of 570 nm [14]. The pH was measured using a MIT65 pH meter. The removal efficiency (*%R*) was calculated based on the following formula [15,16]:

(1) $$\%R=\frac{\left(C_{0}-C_{f}\right)}{C_{0}}100$$

The amount of fluoride adsorbed on LaFeO_3_ NPs, *q_e_*(mg/g) was calculated based on the following formula [17]:

(2) $$q_{e}=\frac{\left(C_{0}-C_{e}\right)V}{M}$$

Where *C*_0_ and *C_e_* are the initial fluoride concentration (mg/L) and equilibrium liquid phase concentration of fluoride (mg/L) respectively; *C_f_* is the final fluoride concentration (mg/L), *V* is the volume of the treated fluoride solution (L) and *M* is the amount of LaFeO_3_ NPs used (g).

## Results and discussion

### SEM, XRD and FTIR analysis on the synthesized LaFeO_3_ NPs

**Fig. 1:**
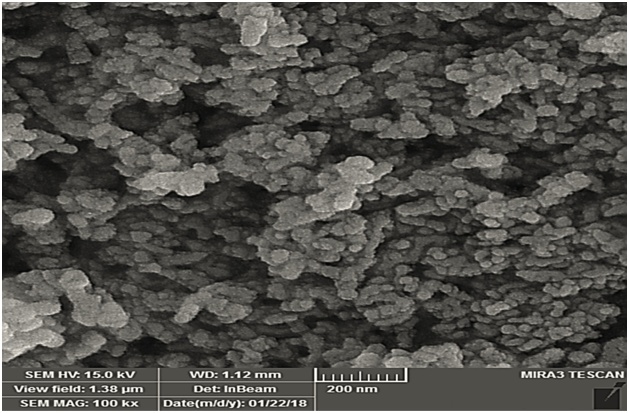
SEM image of synthesized LaFeO_3_ NPs prepared by hydrothermal method.

**Fig. 2:**
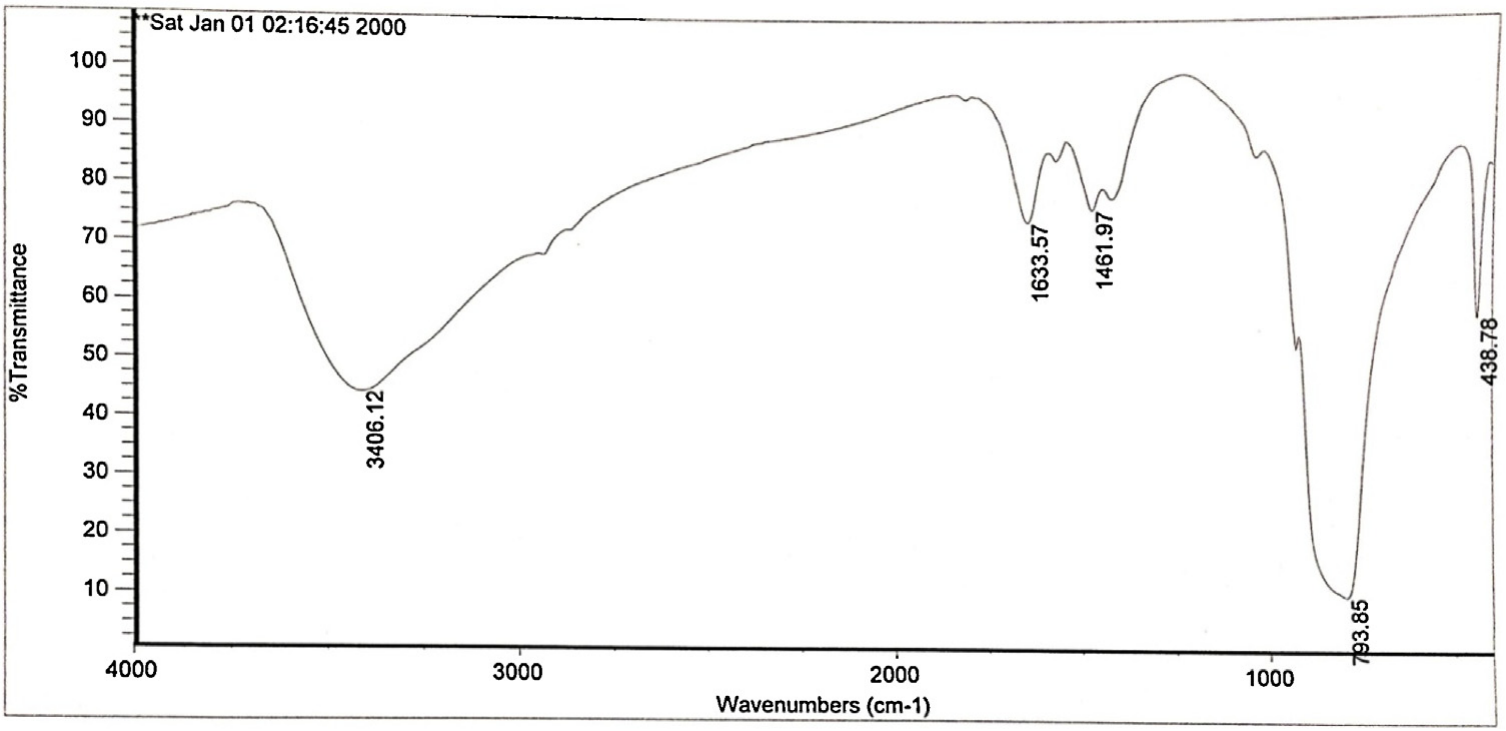
FTIR spectra of the LaFeO_3_ NPs prepared by the hydrothermal method.

The scanning electron microscopy (SEM) image of LaFeO_3_ NPs is shown in Fig. 1. The LaFeO_3_ NPs appears lamellar in arrangement. The surface area of an adsorbent determines its adsorption capability [18]. High porosity was observed on the LaFeO_3_ NPs which indicates that there will be a high level of contact with the fluoride ions [20].The X-ray diffraction (XRD) patterns of LaFeO_3_ NPs is shown in Fig. 2.

The FTIR analysis on theLaFeO_3_ NPs (Fig. 2) indicates the existence of =C—H bend of alkenes (793.85 cm^−1^), and C—H bend of alkanes (1461.97 cm^-1^). The peak 1633.57 cm^-1^ shows the presence of C—C stretch (in-ring) of aromatics. The presence of O—H stretch, H–bonded of alcohols, phenols (3406.12 cm^-1^), which are also strong and broad bands can be observed. The O—H bands are very important sites for adsorption [21]. The hydroxyl group effect is more felt due to the hydrogen bonding with other hydroxyl bonds since they do not exist in isolation establishing a stable structure [21,22].

**Fig. 3:**
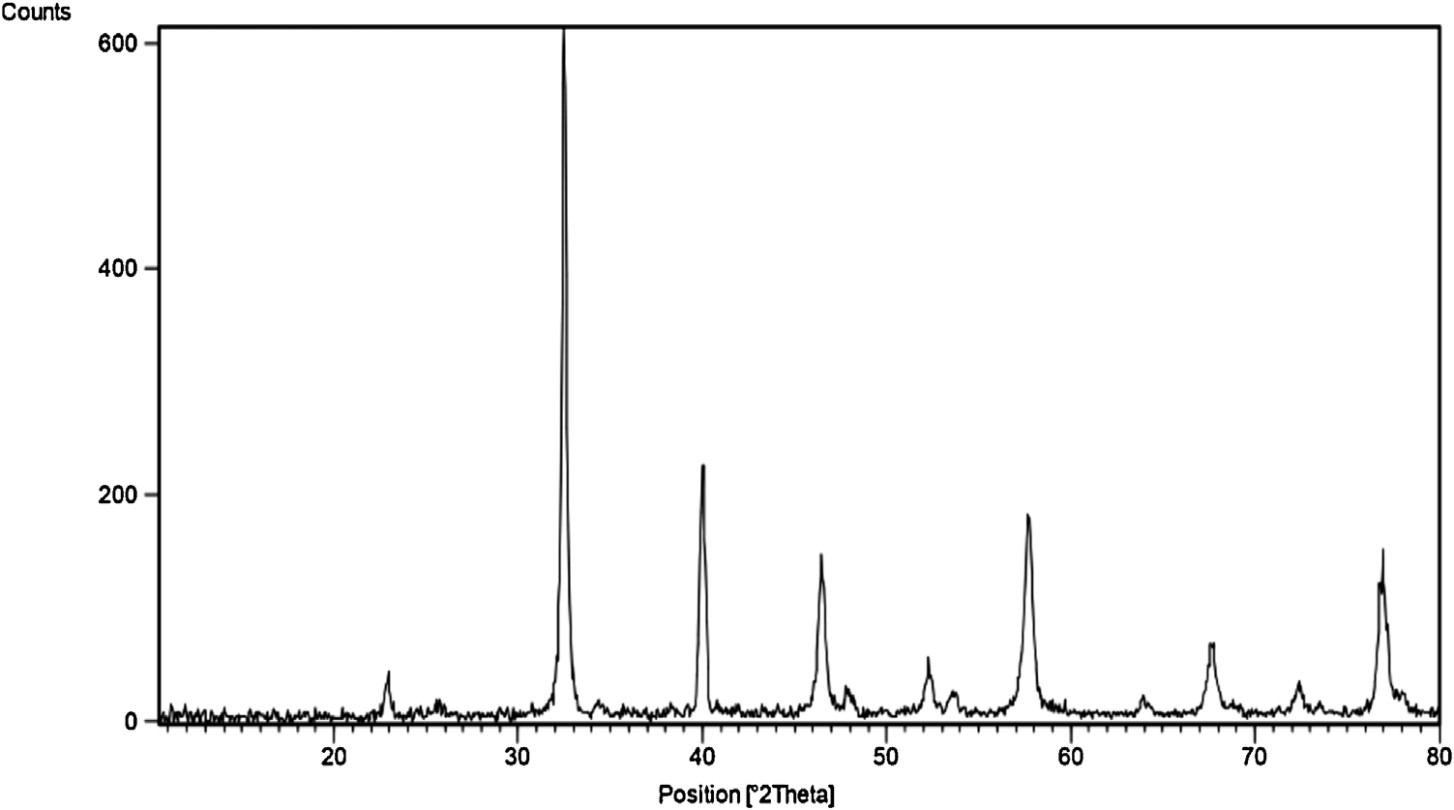
XRD patterns of the LaFeO_3_ NPs prepared by the hydrothermal method.

The XRD result shows that the LaFeO_3_ NPs owns a crystalline structure which improves the process of adsorption by means of physical adsorption [19,20]. Maximum peak of around 2θ = 32.5° (with very high intensity) was also observed on the XRD image (Fig. 3). The average crystallite size (*D*) of LaFeO_3_ NPs nanoparticles was calculated by the Scherer formula (D_h,k,l_ = 0.9λ/β_h,k,l_ cosθ, where λ is the wavelength (1.542 Å), β is the full width at half maximum (FWHM) of the line, and θ is the diffraction angle) [23,24]. The average diameter of the LaFeO_3_ NPs adsorbent (*D*) was calculated to be 35 nm.

### The effect of pH and temperature

**Fig. 4:**
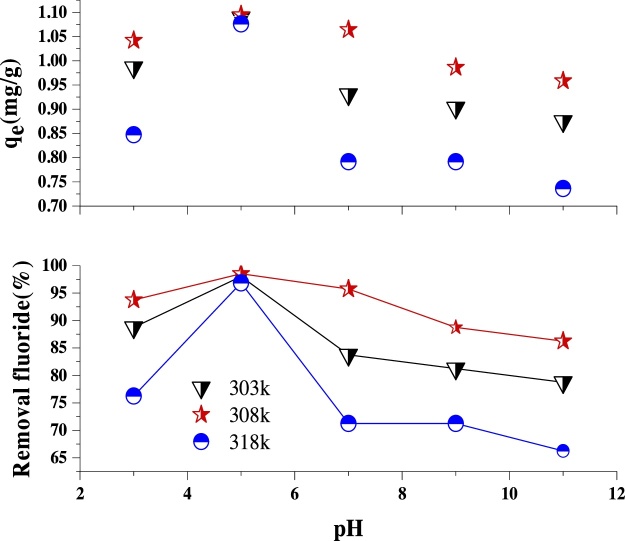
Effect of pH on fluoride adsorption ontoLaFeO_3_NPs (LaFeO_3_NPs dosage: 1 g/L, initial fluoride concentration: 20 mg/L, and contact time: 15 min).

The parameter, pH directly influences the electrostatic interaction between compounds in adsorption processes [25]. To obtain optimal pH value, experiments were carried out by varying the initial pH from 3 to 11, at the constant fluoride concentration of 20 mg/L, LaFeO_3_ NPs dosage of 1 g/L, and contact time of 15 min at different temperatures of 303, 308, and 318 K. The influence of pH with temperature on the percentage removal of fluoride and the amount of fluoride adsorbed (*q*_e_) on LaFeO_3_ NPs surface are shown in Fig. 4. Fluoride reduction was maximum at the temperature of 308 K, which is accepted as the optimum temperature. The temperature increase from 308 to 318 K also allowed the ﬂuorine to desorb to the solution due to damage to the active sites in the adsorbent [26]. Also, it is clear from Fig. 4 that the removal percentage of fluoride increased from 93.75%–98.525 % as the pH was increased from 3 to 5 at the temperature of 308 K but decreased as the pH was increased to 11. Many researchers have reported that the adsorption process is affected by the cationic and anionic forms of the solution due to competition for adsorption among the H^+^ and OH– ions with the adsorbate [27]. In this study, the decrease in the removal efficiency of fluoride as the pH increased may be attributed to electrostatic repulsion [28] between the positively charged LaFeO_3_ NPs and the cationic fluoride. The adsorption of fluoride was more favorable in the acidic environment due to the presence of H^+^ on the adsorbent [29]. The increased amount of H^+^ and reduction of OH– as well as the increase of positive ion can be the reason for the reduction in fluoride removal efficiency on the adsorbent surface [30]. This is also due to the competition of the fluoride ions with excess OH– ions for the adsorption sites at higher adsorption pH [29,30].

### The effect of adsorbent dosage

**Fig. 5:**
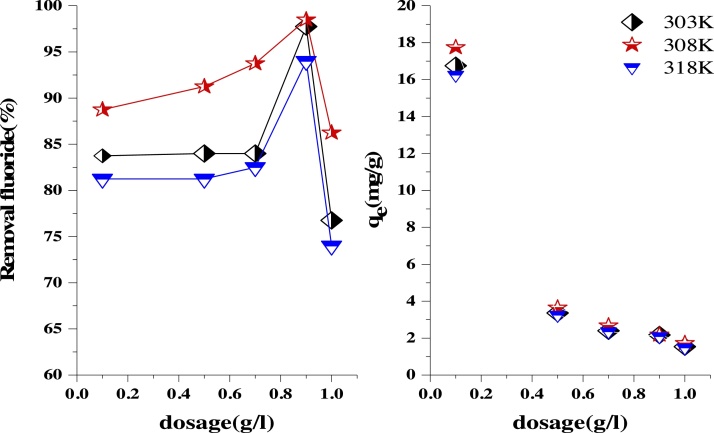
Effect of adsorbent dosage on fluoride adsorption ontoLaFeO_3_ NPs (pH: 5, initial fluoride concentration: 20 mg/L, and contact time: 15 min).

As seen in Fig. 5, the removal of the fluoride increased with increasing the amount of adsorbent (LaFeO_3_ NPs) from 0.1 to 0.9 g/L at different temperatures (303, 308 and 318 K). Maximum fluoride uptake of 98.5 % was observed at an adsorbent dosage of 0.9 g/L and temperature of 308 K. This implies that increasing LaFeO_3_ NPs dose increased the number of active sites available for the adsorption of fluoride. Therefore, the studied adsorbent has a high adsorptive potential, which at very low adsorbent values has a very high uptake of fluoride. With increasing the adsorbent dosages above the optimum (0.9 g/L), the fluoride removal was decreased, which is due to the accumulation of adsorbent particles and the development of electric repulsive force between the adsorbent particles. It can be pointed out that all active sites of the adsorbent were not available to the adsorbate, with this phenomenon being observed more in the batch adsorption process [31].

### The effect of fluoride concentration and contact time

**Fig. 6:**
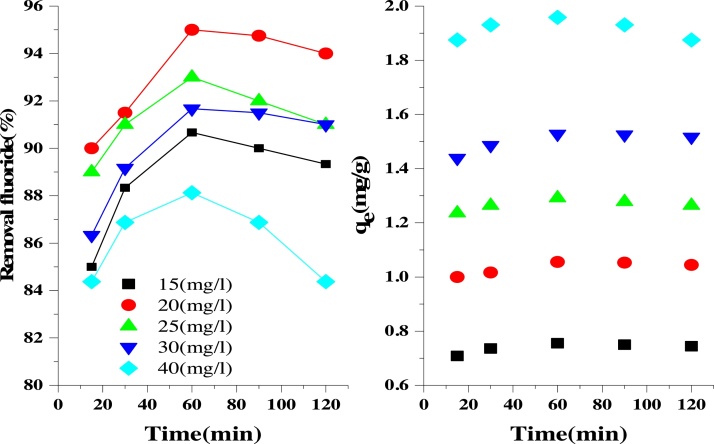
Effect of fluoride concentration and contact time on fluoride adsorption onto LaFeO_3_ NPs (pH: 5, LaFeO_3_ NPs dosage: 0.9 g/L, and temperature =308 K, stirring speed = 150 rpm).

It is important to note that the adsorbate concentration plays a significant role in the removal of pollutants from aqueous solutions and the interaction between the adsorbent and adsorbate species. The effect of concentration on the fluoride adsorption by the LaFeO_3_ NPs was investigated at different initial fluoride concentrations (15, 20, 25, 30, and 40 mg/L) at pH of 5, contact time of 15 min and LaFeO_3_ NPs dosage of 0.9 g/L at different temperatures (Fig. 6). The amount of fluoride adsorbed on LaFeO_3_ NPs (*q_e_*) was increased with increasing fluoride concentration. Also, the percentage of fluoride adsorbed was increased as the initial concentration of fluoride was increased from 15 to 20 mg/L but decreased when the fluoride concentration was increased further at different times of contact. This decrease in efficiency of fluoride removal may be as a result of the over-saturation of the active sites of the adsorbent by the adsorbate [32].

The effect of contact time (15, 30, 60, 90 and 120 min) on the removal of fluoride was studied at pH of 5, LaFeO_3_ NPs dosage of 0.9 g/L, and fluoride concentration of 25 mg/L at different fluoride concentrations (Fig. 6). From Fig. 6 it can be seen that the removal of fluoride increased as contact time increases from 10 to 60 min. Maximum removal of fluoride was achieved in the first 60 min (94.75 %) at the concentration of 20 mg/l. The adsorption of fluoride in the initial minutes was high, including the adsorption rate (Fig. 6) because of reduced fluoride concentration and reduction of the active sites present on the adsorbent surface [33]. The removal efficiency decreased after 60 min because the adsorption sites were occupied [34].

### Sorption kinetics fitting

It is important to emphasize that the optimal contact time is determined based on the adsorption kinetics tests. Kinetic studies are done to observe the mechanism controlling an adsorption process [26]. Kinetic models of adsorption including pseudo-first-order, pseudo-second-order, intraparticle diffusion, fractional and zero-order models were used to test the kinetic data. The equations of kinetic models with the description of the kinetic parameters are stated in Table 1. The kinetic parameters were obtained from the plots of the kinetic models at the optimum conditions of pH 5 and nanoparticles dose of 0.9 g/L. The agreement between the predicted kinetic model values and the experimental data was confirmed by the regression coefficients (r^2^).

**Table 1:** Kinetic models employed to describe the fluoride adsorption by LaFeO_3_ NPs with their respective equations and parameters description [28,29].

Kinetic model	Equation	Parameters description
Pseudo-second-order	$q_{t}=\frac{k_{2p}q_{e}^{2}t}{1+q_{e}k_{2p}t}$	*k*_2_*_p_* = rate constants of the pseudo-second order (g/mg min); *q_e_*= adsorbate amounts at equilibrium (mg/g); *q_t_* = amount of adsorbate removed at time *t* (mg/g).
Pseudo-first-order	$q_{t}=q_{e}\left[1-exp\left(-k_{1p}t\right)\right]$	*k*_1_*_p_*= rate constants of the pseudo- first-order (min^−1^); *q_e_*= adsorbate amount at equilibrium (mg/g); *q_t_* = amount of adsorbate removed at time t (mg/g)
Intra-particle diffusion	$q_{t}=k_{p}t^{0.5}+C$	*q_t_* = amount of adsorbate adsorbed at equilibrium (mg/g); *k_p_* = intraparticle diffusion rate constant (mg/L min^−0.5^); *C* = the intercept which give information about the thickness of the boundary layer
Fractional power	$q_{t}=kt^{v}$	*k*= constant; *v*= rate constant
Zero-order	$q_{t}=q_{e}-k_{0}t$	*q_e_* = adsorbate amounts at equilibrium (mg/g); *k*_0_ =constant

The nonlinear forms of pseudo-first-order, pseudo-second-order, intraparticle diffusion, fractional and zero-order models were used to test the kinetic data. In order to evaluate the validity of the adsorption mathematical kinetic models with the experimental results, a number of error functions are available in the literature. The use of only the regression coefficient (r^2^) for isotherm and kinetic data analysis is not enough, because the experimental results may have high r^2^ values. Therefore, it is necessary to diagnose the result of regression for residue analysis. The applicability of the kinetic model to describe the adsorption process, apart from the regression coefficient (r^2^), was further validated by the normalized standard deviation (NSD), average relative error (ARE) and standard deviation which are defined as Eqs. (3), (4), (5), respectively:

(3) $$\text{NSD}=100\sqrt{\frac{1}{N-1}\sum_{i=1}^{N}{\left[\frac{q_{t}^{\exp}-q_{t}^{cal}}{q_{t}^{\exp}}\right]}^{2}}$$

(4) $$ARE=\frac{100}{N}{\sum_{i=1}^{N}\left|\frac{q_{e}^{\exp}-q_{e}^{cal}}{q_{e}^{\exp}}\right|}_{i}$$

(5) $$SD=\sqrt{\left(\frac{1}{N-P}\right)\sum_{i=1}^{N}{\left(q_{i,observed}-q_{i,eale}\right)}^{2}}$$

Where *N* is the number of performed experiments, *P* is the number of parameters of the fitted model, and r^2^ is the coefficient of determination; *q_t_^exp^* and *q_t_^cal^* are the experimental and calculated amounts of fluoride adsorbed on LaFeO_3_ NPs at time t (mg/g). The model with the highest values of r^2^ and the lowest values of SD best represents the process. The smaller NSD and ARE values indicate a more accurate estimation of *q_t_* values [20,25].

The nonlinear adsorption kinetics results obtained are presented in Table 2. It is observed that the pseudo-second-order kinetic model best described the kinetic experimental data with the value of r^2^ closer to unity (0.8577). The pseudo-second-order possesses lower values of NSD (0.8873), ARE (0.7120), and SD (0.0106) when compared with the other kinetic models. This means that the fluoride adsorption onto LaFeO_3_ NPs is a chemical type of adsorption [35]. This also indicates that the calculated values of *q_t_* (*q_t_^cal^*) obtained from the pseudo-second-order model extremely correspond with the experimental values of *q_t_* (*q_t_^exp^*).

**Table 2:** Nonlinear kinetic parameters for adsorption of fluoride onto LaFeO_3_ NPs at optimal condition (pH: 5, nanoparticles dose: 0.9 g/L, temperature =308 K).

Model		r^2^	NSD	ARE(%)	SD
**Pseudo-second-order**		0.8577	0.8873	0.7120	0.0106
$k_{2p}$	0.9922				
$q_{e}$	1.06				
**Pseudo-first-order**		0.6422	1.4232	0.9592	0.0169
$k_{1p}$	1.043				
$q_{e}$	0.2088				
**Intra-particle diffusion**		0.7108	1.2542	0.9498	0.0152
$k_{p}$	0.007144				
C	0.9803				
**Fractional power**		0.7991	1.0431	0.7734	0.0126
k	0.9377				
v	0.02502				
**Zero-order**		0.5977	1.4849	1.1560	0.0179
$k_{0}$	−0.0004393				
$q_{e}$	1.006				

### Equilibrium isotherms and fit error evaluation

In different adsorption investigations, the study of the adsorption of pollutants on the surface of adsorbents, determining the adsorption capacity (*q_m_*) and adsorption isotherm models that best fit the experimental data are of great importance to many researchers. The way a pollutant is adsorbed on an adsorbent can be interpreted through the study of adsorption isotherms. Isotherms can represent the relationship between the pollutant concentration present in the solution and the amount of pollutant adsorbed by the solid phase when both phases are at equilibrium. The equations of isotherm models with the description of their parameters are stated in Table 3. The correlation coefficient (r^2^) is used to judge whether experimental data follow isotherm models [36]. In addition to r^2^, the parameters of average relative error (ARE), Marquardt’s percent standard deviation (MPSD) and Hybrid error function (HYBRID), root mean squared error (RMSE), and normalized standard deviation (Δ*q(%)*) were also evaluated, which can be described as Eqs. (4), (6), (7), (8), (9), respectively:

(6) $$MPSD=100\sqrt{\frac{1}{N-P}\sum_{i=1}^{N}{\left[\frac{q_{ei}^{\exp}-q_{ei}^{cal}}{q_{ei}^{\exp}}\right]}^{2}}$$

(7) $$HYBRID=\frac{100}{N-P}\sum_{i=1}^{N}{\left[\frac{q_{ei}^{\exp}-q_{ei}^{cal}}{q_{ei}^{\exp}}\right]}^{2}$$

(8) $$RMSE=\sqrt{\frac{1}{p-2}\sum_{i=1}^{p}{\left(q_{e}-q_{c}\right)}^{2}}$$

(9) $$\Delta q\left(\%\right)=100\sqrt{\frac{\sum {\left|\left(q_{ei}^{exp}-q_{ei}^{cal}\right)/q_{ei}^{exp}\right|}^{2}}{N-1}}$$

Where *q_ei_^exp^* is the observation from the batch experiment *i*, *q_ei_^cal^* is the estimate from the isotherm for the corresponding *q_ei_^exp^*, *N*is the number of observations in the experimental isotherm and *p* is the number of parameters in the regression model; *q_c_* is the value that is calculated from model fit and *q_e_* is calculated from test elements. The smaller MPSD and HYBRID values indicate a more accurate estimation of *q_e_* value [20]. MPSD and HYBRID functions were used in addition to r^2^ because the number of parameters in the regression model (that is, *p* parameter) is effective in them.

**Table 3:** Isotherm models employed to describe the fluoride adsorption by LaFeO_3_ NPs with their respective equations and parameters description [[37], [38], [39]].

Isotherm model	Equation	Parameters description
Langmuir	$\frac{C_{e}}{q_{e}}=\frac{C_{e}}{q_{m}}+\;\frac{1}{q_{m}K_{L}}$	*K_L_* = Langmuir constant (L/mg); *q_e_* = amount of adsorbate adsorbed (mg/g); *q_m_* = maximum/monolayer adsorption capacity (mg/g); *C_e_* = equilibrium concentration of adsorbate in solution (mg/L)
Freundlich	${q_{e}=K}_{F}C_{e}^{\frac{1}{n}}$	*K_F_* = Freundlich constant; *q_e_* = amount of adsorbate adsorbed (mg/g); *C_e_* = equilibrium concentration of adsorbate in solution (mg/L), *n* = intensity of adsorption
Temkin	${{\text{q}}_{\text{e}}=\text{B}}_{1}\text{l}\text{n}\left({\text{A}}_{\text{T}}\right)+\text{l}\text{n}\left({\text{C}}_{\text{e}}\right)$	*C_e_* = equilibrium concentration of adsorbate in solution (mg/L) ; *q_e_* = amount of adsorbate adsorbed (mg/g); *B*_1_ = heat of sorption; *A_T_*= equilibrium binding constant
Koble–Corrigan	$q_{e}=\frac{A_{KC}{\left(C_{e}\right)}^{P}}{1+B_{KC}{\left(C_{e}\right)}^{P}}$	*C_e_* = equilibrium concentration of adsorbate in solution (mg/L); *q_e_* = amount of adsorbate adsorbed (mg/g); *B_KC_*, *A_KC_*and*p* = Koble–Corrigan isotherm constants
Redlich–Peterson	$q_{e}=\frac{A_{RP}\left(C_{e}\right)}{1+B_{RP}{\left(C_{e}\right)}^{g}}$	*C_e_* = equilibrium concentration of adsorbate in solution (mg/L); *q_e_* = amount of adsorbate adsorbed (mg/g); A_RP_=Redlich–Peterson isotherm constant (L/g);*B_RP_*_=_ constant (L/mg), and *g* = exponent that lies between 0 and 1.
Dubinin–Radushkevich	$q_{e}=q_{D}exp\left(-D\varepsilon^{2}\right)$ $\varepsilon =RTln\left(1+\frac{1}{C_{e}}\right)$	*C_e_* = equilibrium concentration of adsorbate in solution (mg/L); *q_D_* = theoretical saturation capacity (mg/g) ; *q_e_* = amount of adsorbate adsorbed (mg/g); *D* = constant related to mean free energy of adsorption per mole of the adsorbate (mol^2^/J^2^); *R*= universal gas constant (8.314 J/mol/K) ; *ε* = polanyi potential

**Fig. 7:**
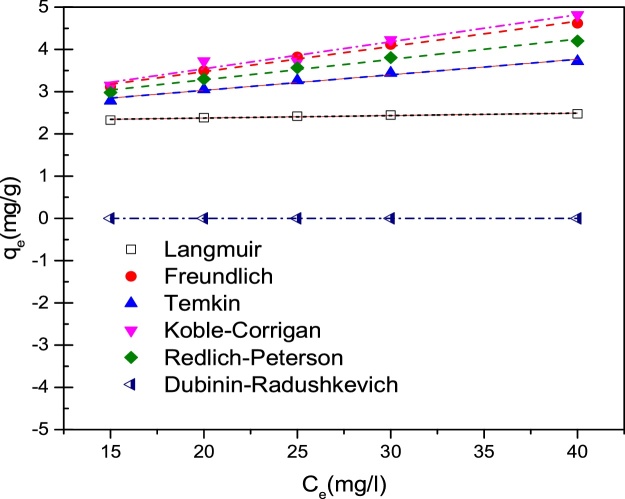
Isotherm plots for fluoride adsorption by LaFeO_3_ NPs (Adsorption conditions: pH = 5, LaFeO_3_ NPs dose = 0.9 g/L, stirring speed = 150 rpm, temperature =308 K).

The isotherm plots for fluoride adsorption by LaFeO_3_ NPs at the optimum conditions of pH 5, LaFeO_3_ NPs dose of 0.9 g/L, and temperature of 308 K is shown in Fig. 7. Considering the r^2^ values obtained for the theoretical models evaluated (Table 4), it can be observed that the six isotherm models (Langmuir, Freundlich, Temkin, Koble–Corrigan, Redlich–Peterson and Dubinin–Radushkevich) present firm adherence to the experimental data. This shows the good agreement between the calculated *q_e_* and the experimental *q_e_* values for all isotherms. By evaluating the values of all the error functions applied to the adjusted models, the Freundlich and Koble–Corrigan models were the most suitable to describe the observed phenomenon. Its fit into the Freundlich model suggests a heterogeneous and multilayer adsorption of the fluoride on the LaFeO_3_ NPs surface. Since the Freundlich model describes a chemical adsorption process, it supports the kinetic approach which denoted a chemical behavior of the adsorption of fluoride on LaFeO_3_ NPs. The monolayer adsorption capacity of LaFeO_3_ NPs (*q_m_*) was obtained as 2.575 mg/g. An adsorption intensity (*n*) value of 2.488, which is within 1–10 (1 <*n*< 10) obtained for the fluoride adsorption proposes that the adsorption process on LaFeO_3_ NPs is favorable [20].

**Table 4:** Isotherms parameters provided by isotherm models, with the error functions evaluated for sorption of fluoride by LaFeO_3_ NPs.

Model		r^2^	MSPD	HYBRID	RMSE	ARE (%)	Δq(%)
**Langmuir**		0.9788	5.7628	0.3988	2.48E-03	3.6762	22.1396
$q_{m}$	2.575						
$K_{L}$	0.6225						
**Freundlich**		0.9985	1.3841	0.0250	1.66E-04	0.8230	10.4752
$K_{F}$	1.048						
*n*	2.488						
**Temkin**		0.9870	4.2127	0.2222	1.44E-03	2.7356	19.0982
$B_{1}$	0.5859						
$A_{T}$	5.644						
**Koble–Corrigan**		0.9985	1.9102	0.0487	1.64E-04	0.8080	10.3792
$A_{KC}$	1.02						
P	0.3862						
$B_{KC}$	−0.02757						
**Redlich–Peterson**		0.9976	2.7400	0.0896	2.71E-04	1.0819	12.0104
$A_{RP}$	6.216						
*g*	0.666						
$B_{RP}$	4.998						
**Dubinin–Radushkevich**		0.8724	12.1894	2.0137	1.44E-02	8.4500	33.5659
$q_{D}$	1.921						
D	2.237E-07						

### Thermodynamic studies

Temperature has a great impact on the adsorption process, so the thermodynamic study. The thermodynamic parameters including the standard Gibbs free energy (ΔG°), enthalpy change (Δ*H*°), and entropy change (Δ*S*°) are useful in defining whether the sorption reaction is endothermic or exothermic, and spontaneity of the adsorption process. The thermodynamic parameters (ΔG°, Δ*H*°, and Δ*S*°) for adsorption of fluoride onto LaFeO_3_ NPs were calculated (Table 5) using the following equations [20]:

(10) $$\Delta G^{0}=-\text{RTln}{\text{K}}_{\text{a}}$$

(11) $$\Delta G^{0}=\Delta {\text{H}}^{0}-\text{T}\Delta {\text{S}}^{0}$$

Where, *R* is the universal gas constant (8.314 J/mol/K) and *T* is the absolute temperature in K.

**Table 5:** Thermodynamic parameters for the adsorption of fluoride using LaFeO_3_ NPs.

Temperature (K)	*C*_0_ (mg/L)	*ΔS*° (kJ/mol.K)	*ΔH*° (kJ/mol)	*ΔG*° (kJ/mol)
298				−3.94
308	20	−0.0115	−0.5057	−4.06
318				−4.17

**Fig. 8:**
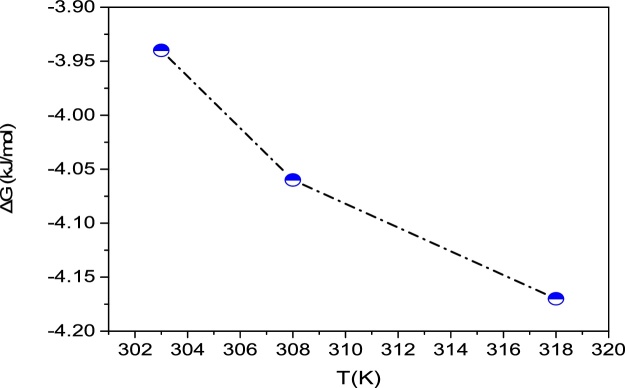
The plot of Gibbs free energy change, ΔG°, versus temperature, *T*.

The thermodynamic parameter, Gibb’s free energy change (ΔG°) is calculated using *K_a_* obtained from the Langmuir isotherm. The values of Δ*H*° and Δ*S*° were evaluated from the intercept and slope of the regression plot of ΔG° versus *T* (Fig. 8).

All the values of ΔG° were negative; this shows that the fluoride adsorption process by LaFeO_3_ NPs was spontaneous (ΔG°< 0) and feasible [40]. The decreased amount of ΔG° with the increase in temperature indicates that the increase in temperature resulted in an increase in spontaneity. The negative ΔH° value of adsorption reaction on LaFeO_3_ NPs (−0.5057 kJ/mol) indicated that the process was exothermic (Δ*H*°< 0) [41]. According to Le Chatelier's principle, increasing the temperature reduced the reaction rate. Entropy change (Δ*S*° = -0.0115 kJ/mol.K) of fluoride adsorption by LaFeO_3_ NPs is negative, suggesting that the degree of freedom at solid-solution level declines during the adsorption [30]. The negative value of Δ*S*° may be caused by the decrease in the efficiency of the reaction with higher temperatures [42,43].

### Comparison of LaFeO_3_ NPs with other adsorbent materials on fluoride removal

The removal of fluoride on LaFeO_3_ NPs was compared with other adsorbent materials employed by several authors in terms of percentage removal efficiency (Table 6). The fluoride removal efficiency of 94.75 % obtained using LaFeO_3_ NPs indicates that it can be applied for fluoride removal from its aqueous solution. Generally, the results obtained by the authors shown in Table 6 show that the different adsorbents can be harnessed for the removal of fluoride via the adsorption process.

**Table 6:** Comparison of LaFeO_3_ NPs with other adsorbents for fluoride reduction.

Adsorbent material	Maximum removal (%)	Conditions	Reference
Nickel oxide nanoparticles	98.75	pH = 5; Adsorbent dosage =0.02 g; Initial concentration = 20 mg/L; Time =60 min; Volume of fluoride solution, V = 100 mL; Temperature =298 K ; Speed = 150 rpm	[44]
Synthesized P/γ-Fe_2_O_3_ nanoparticles	99	pH = 7; Adsorbent dosage =0.02 g/L; Initial concentration =25 mg/L; Time =30 min; V = 1 L; Speed = 150 rpm	[14]
Chitosan	87	pH = 7; Adsorbent dosage =5 g/L; Initial concentration =5 mg/L; Time = 180 min	[45]
Modified Turkish zeolite with quaternary ammonium	85	pH = 5; Adsorbent dose = 20 mg/L; Contact time =60 min; Temperature =293 K; Initial ﬂuoride concentration = 10 mg/L V = 100 mL; Stirring speed = 200 rpm	[26]
Peanut husk	82.3	pH = 3; Adsorbent dosage =6 g/L; Initial concentration = 10 mg/L; Time =80 min; V = 100 mL; Temperature = 23 ± 2 °C	[46]
Lanthanum ferrite nanoparticles (LaFeO_3_ NPs)	94.75	pH = 5; LaFeO_3_ NPs dosage = 0.9 g/L; Fluoride concentration = 20 mg/L; Time =60 min; V = 100 mL; Temperature =308 K; Speed = 150 rpm	This study

## Conclusion

The removal of fluoride on lanthanum ferrite nanoparticles (LaFeO_3_ NPs) was found to be dependent on the initial pH, temperature, dosage of LaFeO_3_ NPs, contact time, and initial fluoride concentration. Under optimal conditions of fluoride concentration of 20 mg/L, pH of 5, LaFeO_3_ NPs dosage of 0.9 g/L, temperature of 308 K, and contact time of 60 min, maximum percentage removal of 94.75 % was obtained. Adsorption kinetics, isotherm, and thermodynamics were studied for ﬂuoride ions removal on LaFeO_3_ NPs. The monolayer adsorption capacity of LaFeO_3_ NPs was 2.575 mg/g. The adsorption process fitted well into the Freundlich, Koble–Corrigan and pseudo-second-order kinetic models considering the values of the regression coefficients (r^2^) and error functions used. The fluoride adsorption on LaFeO_3_ NPs was found to be favorable, exothermic and spontaneous in nature. Its spontaneity was increased with temperature. From the study, it can be concluded that the LaFeO_3_ NPs prepared by the hydrothermal method can be used for the effective reduction of fluoride concentration in aqueous environments. Since simulated fluoride effluent was used in the present study, further studies can be carried out on real fluoride-containing wastewater.

## Funding sources

The authors thank the Research Assistance of Zabol University of Medical Sciences (No. IR.ZBMU. REC.1396.330) for financial and spiritual.

## Declaration of Competing Interest

The authors declare that they have no competing financial interests or personal relationships that could have appeared to influence the work reported in this paper.
